# To adopt or adapt an existing neonatal core outcome set in Kenya: a study protocol

**DOI:** 10.1186/s13063-023-07821-z

**Published:** 2023-12-15

**Authors:** Jamlick Karumbi, David Gathara, Bridget Young, Paula Williamson

**Affiliations:** 1 Department of Health Data Science, University of Liverpool, Liverpool, UK; 2 Health Systems Research, KEMRI-Wellcome Trust Research Programme, Kilifi, Kenya; 3 Centre for Maternal, Adolescent, Reproductive & Child Health (MARCH), London School of Hygiene and Tropical Medicine, London, UK; 4 Department of Public Health, Policy and Systems, University of Liverpool, Liverpool, UK

## Abstract

**Background:**

Development and use of core outcome set(s) (COS) in research can reduce research wastage by ensuring that a minimum set of outcomes are always reported on. Neonatal morbidity and mortality are a big burden in low- and middle-income countries (LMICs). Research is continuously being undertaken to reduce this burden. Currently, there is no COS for neonatal research in LMICs but there exists one for neonatal research in high-income countries (HICs).

**Objectives:**

To determine outcomes that are useful for neonatal care in Kenya to inform whether an existing COS should be adopted or adapted. To assess the feasibility of a routine data collection system to collect data of the agreed-on COS.

**Methods:**

A review of existing literature on neonatal research in LMICs followed by a qualitative study of key stakeholders in neonatal care. To explore whether to adapt or adopt, in two hospitals, two focus group discussions with 6–8 parents/caregivers will be undertaken (one each in two hospitals). Key informant interviews will also be conducted with 6 health care providers in each of the hospitals. At the policy-making level, we will conduct 10 key informant interviews. Qualitative data will be analyzed thematically. A consensus meeting will be undertaken with key stakeholders, who will be presented with an overview of the COS developed for HICs, key findings from the literature, and the qualitative study to determine context-appropriate COS. The agreed-on outcomes will be counterchecked against the case records in the two hospitals. The feasibility of collecting the outcomes on a routine electronic research database, the Clinical Information Network that collects standardized data at admission and discharge, will be explored. The congruence (or not) of the outcomes will be documented and be used to enrich the discussion and provide a snapshot of the feasibility of the health information system to collect routine data on the COS.

**Conclusions:**

A COS for use in neonatal care in Kenya will help enhance outcome measurements and reporting not just in research but also in routine practice. This will enhance the comparability of interventions in trials and routine settings leading to reduced research wastage and likely improved quality of care. Additionally, the methodology used for this work can be adopted in other settings as a means of adopting or adapting an existing COS.

**Supplementary Information:**

The online version contains supplementary material available at 10.1186/s13063-023-07821-z.

## Background

Even though there has been a substantial decline in the deaths of infants and young children in the last two decades, the burden of disease and mortality remains high [1]. In 2019, almost half, two and half million of deaths in young children occurred during the first month of life, (neonatal period; 0–28 days), with the vast majority (> 90%) occurring in low- and middle-income countries (LMICs) [2]. The most common causes of these neonatal deaths are prematurity, intrapartum complications, and neonatal sepsis.

In Every Newborn Action Plan (ENAP) of 2014 and Sustainable Development Goal 3.2, countries made a commitment to reduce the neonatal mortality rate to at least as low as 12 deaths per 1000 live births by 2030 [3, 4]. For this to be achieved, countries need to scale up coverage and implementation of evidence-based high-impact interventions (Kangaroo mother care, treatment of small for gestational age and sick neonates) [5]. Quality data is required to keep track of this coverage and implementation. There are challenges with neonatal care data in LMICs, for example, data is not readily available from routine health information systems [mainly collected in health surveys] and where available, the quality is poor due to a lack of standardized registers across various hospitals and non-linkage of datasets within and across hospitals [6].

A 2015 study by Aluvaala and others estimated the main causes of admission in 22 Kenyan County hospitals (Formerly District hospitals) to be birth asphyxia (36%), prematurity/low birth weight (32%), and neonatal sepsis (19%) [7]. There have been improvements in newborn health with recent estimates across 16 hospitals in Kenya showing the main causes of newborn admission as intrapartum-related complications (30%), respiratory distress syndrome (18%), neonatal sepsis (15%), jaundice (12%), and uncomplicated low birth weight (LBW)/prematurity (5%) [8]. In both of these studies, comorbidities were common, and the newborns were on multiple interventions as part of newborn care within the newborn admission ward/newborn unit.

Care for neonates in Kenya is guided by policies made at the national level (National government) and service provision by County governments (public sector) and other private and non-governmental health facilities. Despite infrastructural and capacity investment for neonatal care in Kenya over the last few years, neonatal deaths accounted for two thirds of children admitted in the 16 hospitals with more than half of these deaths occurring within 24 h of admission [8]. There is a need to continuously undertake research on how to enhance the quality of care during this neonatal period. One of the key undertakings by policy makers is to develop clinical practice guidelines and data collection tools and registers that guide neonatal service provision. Kenya has adopted the GRADE (Grading of Recommendations Assessment, Development and Evaluation) approach to the development of pediatric clinical guidelines. This approach is heavily reliant on comparable outcomes across studies to allow for proper evidence synthesis. During the last revision of the Kenyan pediatric protocols (which includes neonates), in the review of evidence on neonatal care, heterogeneity of outcome data on neonatal sepsis was noted as a key limitation to conducting a meta-analysis, leading to policy makers depending on low to moderate quality of evidence for the recommendation on umbilical cord care [9]. There was also a scarcity of routine health system data on neonatal care. To enhance research synthesis for clinical guidelines and enable setting up of pragmatic trials, outcomes that are reported in neonatal care and research need to be comparable [10].

One of the ways to achieve this comparability of outcomes in neonatal care is to ensure that we have standardized outcomes to guide neonatal research and clinical care in LMICs.

## What are COS?

A core outcome set (COS) is an agreed-on minimum standardized outcome set(s) that should be measured and reported in all research in a given health area [11]. A COS consists of a core domain set (this defines what domains should be measured in a trial) and core measurement tools (defining how the outcomes should be measured). Core outcomes are important outcomes agreed on by key stakeholders (including patients or public participants) using robust consensus-building methods. The use of COS could ensure all future research and routine systems collect not only clinically important outcomes but also help to ensure the inclusion of outcomes that are important to patients/caregivers and policy formulators. This could reduce research waste, by facilitating meta-analysis, minimizing reporting bias, and thereby enhancing research translation and use [12]. COS are also becoming useful in routine data collection systems, which enhances designing the quality of neonatal care initiatives through quality audits and feedback [13, 14]. It is worth noting that since a COS is a minimum set, it does not limit researchers and clinicians reporting other outcomes which they may deem relevant. COS use is becoming increasingly recommended by research funders, journal editors, and clinical guideline developers in high-income countries (HICs) with the potential to reduce research waste and improve the quality of research reporting [15].

### COS development

Globally, COS have been developed for various conditions or diseases and continue to be developed [16, 17]. There has been an increase in the inclusion of LMIC stakeholders in COS development and use. However, most COS work has been undertaken in HICs with only 20% of the 370 COS registered in the COMET Initiative database having stakeholders from LMICs. Only four COS have been initiated from LMICs [18]. LMICs often have different burdens of disease, health care systems, resources, and research infrastructure. This could in effect mean that the COS identified in a HIC setting for a given disease area may not necessarily be applicable in LMICs.

Some of the reasons cited for low initiation of COS and inclusion of stakeholders from LMICs are lack of knowledge on COS availability and utility, health system issues like lack of tools or means to measure the agreed-on outcomes, and inadequate funding to fully engage stakeholders [19]. It is also postulated that literacy levels, use of technology, and lack of formal patient organizations or associations could reduce the involvement of patients or lay public though optimal patient involvement strategies are still being developed [20].

### Transferability of COS developed in HICs to LMICs

It is increasingly realized that to make research more relevant to practice, contextualization is of great importance. Encouraging and normalizing the development and use of relevant COS by researchers, clinicians, funders, and policy makers is a key priority to make the COS more globally applicable. As noted above, LMIC stakeholder inclusion has expanded over the last few years of COS development to include more patients, caregivers, and lay public. There has been a cross-linking of COS for research with clinical practice with the aim of using the COS in clinical audit and feedback for improvement of care [21]. There is however paucity of evidence as to whether the methods for COS development that have worked in HICs can be used in LMICs and whether a COS developed in a HIC can be used as is in an LMIC or it would require adaptation.

Only one COS has been led from an African setting. This is a COS on congenital abnormalities as part of setting up a congenital abnormalities’ registry in Rwanda [22]. However, as of the end of 2019, of the 370 COS registered in the COMET database, 22 had stakeholders from African countries though they were mainly drawn from South Africa [18]. No COS has been initiated in Kenya.

### Why neonatal COS?

With a current nationally estimated 20 deaths per 1000 live births in the neonatal period, Kenya has a long way to go if the target of at least as low as 12 deaths per 1000 live births is to be achieved [23]. Even though there is an improvement in facility-based deliveries and by extension care of newborns and neonates, based on admission data spanning 1 April 2018 to 31 March 2020 from 16 hospitals, neonatal deaths still account for two thirds of pediatric admissions deaths in Kenya [8]. Improvement of neonatal care needs to be enhanced and multipronged. For this to happen, the right quality data is needed to ensure that there are targeted interventions. One of the challenges faced by policy makers and clinicians getting the right quality data is the lack of standardized outcomes being reported not just for routine newborn care but also for neonatal research. One of the ways to have these standardized outcomes is to have a minimum set of outcomes being reported in routine practice and research. This minimum set needs to be agreeable to the key stakeholders in the local context for implementation to be efficient.

### Neonatal care COS

There exists a COS for neonatal care that was developed in 2019 by Webbe and others [24]. The study set out to define a core outcome set (COS) for research involving infants receiving neonatal care in a high-resource setting. The process included a three-round e-Delphi survey^1^ with 173 participants and a face-to-face consensus meeting of 16 people, to confirm the final COS based on the survey results. The participants were former patients cared for in a neonatal unit, and parents of neonatal patients, doctors, nurses, and researchers.

While some stakeholders from LMICs were included in the development process, their inclusion was based on their prior experience of neonatal care or research in a high-resource setting neonatal unit. Twelve outcomes were included in the final COS: survival, sepsis, necrotizing enterocolitis, brain injury on imaging, general gross motor ability, general cognitive ability, quality of life, adverse events, visual impairment/blindness, hearing impairment/deafness [for all neonates], retinopathy of prematurity and chronic lung disease/bronchopulmonary dysplasia [for preterm babies only]. The study did not provide recommendations for how these outcomes should be measured.

Given that this COS was developed from a high resource perspective, it is unclear whether the COS may be directly transferable into LMIC settings due to several reasons:

The differing resource availability for running neonatal care units may mean that the quality of care provided and therefore positive outcomes are more likely in HICs compared to LMICs, and therefore, the outcomes important to parents in HIC settings might differ from those of parents in LMICs.

Some of the outcomes suggested for the COS may already be being measured routinely which may not be the case in LMICs (for example imaging in neonates) [25].

The differing epidemiology of neonatal diseases, for example in LMICs there are a number of underlying comorbidities, may mean neonates would be given several interventions at the same time and this may lead to different outcomes in this setting.

Due to the availability of systems to capture information on the neonates, enabling follow-up of patients, the authors of the HIC COS study were able to obtain opinions of former neonatal unit patients. This follow-up information is often lacking in LMICs. Indeed, HIC systems are more able to capture long-term outcomes compared to LMICs where short-term outcomes may be more likely to be identified.

In the development of this COS, policy makers and hospital administrators were not included as participating stakeholders. It is possible that, when this group of stakeholders is involved, the set of outcomes that are useful to them might be different. Policy makers determine indicators being collected by the routine health system and determine resource allocation which affects data collection and availability (adequacy of health workforce, availability of standard registers tools, etc.).

There is a need to demonstrate the feasibility (or not) of either adopting or adapting a COS for neonatal care and research developed in a HIC setting for use in an LMIC setting. This work is important as it will help describe a methodology that can be applied to other COS that have had low participation from LMIC stakeholders but are important in LMIC settings.

In this study, we will engage key stakeholders, parents/caregivers of neonates, clinicians managing neonates in newborn units, researchers undertaking neonatal research, and policy makers responsible for newborn and child-health policies in Kenya to understand if neonatal care outcomes identified for a HIC setting should be measured (adopted) or would require adaptation in a low resource setting. Additionally, we will assess the feasibility of the routine health information system to collect data for the neonatal care COS.

### Rationale

Even though a COS for neonatal care exists, it was developed for use in HIC newborn care [24]. Further, stakeholders that were involved in the neonatal care COS for HICs were mainly LMIC researchers and clinicians who had experience in neonatal care or research in HIC. There were no policy makers or hospital managers involved in that process.

It is important to document the process of adapting an existing neonatal care COS and describe the contextual issues that need to be addressed for the adaptation to be successful.

We propose to use qualitative inquiries and a face-to-face consensus-building process in determining contextual issues that are important for the various stakeholders in Kenya to ensure that all important outcomes are measured and reported in neonatal care research and practice. Once these outcomes are agreed upon, it is useful that we assess whether a routine health system is already collecting the data or is capable of collecting this data. Since this work is constrained within a PhD timeline, it may not be feasible to use the routine Kenya Health Information System. We will therefore use the Clinical Information Network (CIN) to assess this. CIN brings together 22 county hospitals that form the first line of referral in Kenya. It promotes the generation and use of high-quality routine information on hospital admissions to pediatric and neonatal wards as part of learning health systems [26]. CIN uses these data to promote and track adherence to guidelines and provides 3-monthly audit and feedback reports on key quality of care indicators as a means of quality improvement [27].

## Aim

The overall objective of this work will be to determine outcomes that are useful for neonatal care in Kenya to inform whether an existing COS should be adopted or adapted and assess the feasibility of a routine data collection system to collect the COS data.

## Specific objectives

1. To identify outcomes being reported in neonatal care research conducted in sub-Saharan Africa through a rapid review of available literature.
2. To explore the important outcomes for key stakeholders (parents/caregivers of children who are under neonatal care, clinicians, researchers, and policy makers) in Kenya.
3. To undertake a consensus-building process on the important outcomes for neonatal care in Kenya.
4. To assess the feasibility of collecting the proposed COS data using a routine data reporting system (CIN).

## Methodology

### Scope

The COS will include the most relevant outcome measures for neonatal care research and clinical practice in Kenya for all stakeholders involved in neonatal care and research (first 28 days of life). This COS will be designed to standardize the reporting of outcomes in neonatal care research and clinical practice.

### Design

**Fig. 1:**
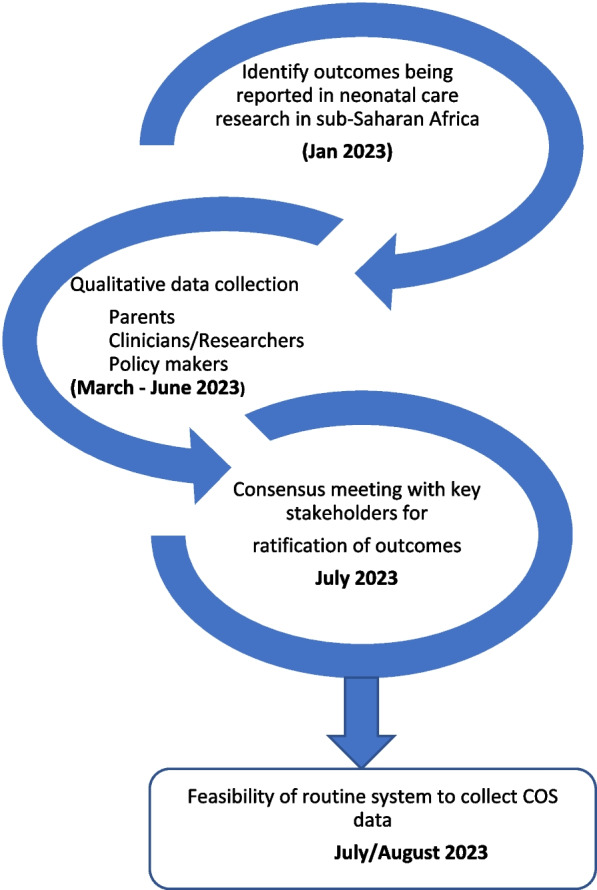
Neonatal care COS adoption/adaptation process

This section is guided by the published recommendations in the COMET handbook [28] and will involve steps as shown in Fig. 1. The initial step will be to undertake a rapid review on the outcomes being reported for neonatal care research in sub-Saharan Africa. A search strategy will be developed with the following key terms (neonatal care OR newborn care OR neonatology) AND (clinical trials OR randomized clinical trials). Since the aim is to identify outcomes that are relevant to the Kenyan setting, the search will be limited to research undertaken in sub-Saharan countries and in the English language. The outcomes reported in the identified trials will be categorized as short-term (outcomes occurring in the neonatal period [first 28 days of life]) and long-term (outcomes in the post-neonatal period) and combined with the outcomes reported in the HIC COS.

During the qualitative studies (key informant interviews, focus groups) described in objective two, different groups will be asked to rate the identified outcomes as part of identifying outcomes that are important to stakeholders in Kenya.

A final face-to-face consensus meeting will be undertaken to reach an agreement on the COS for neonatal research in LMICs.

We settled on using a qualitative approach as it provides an opportunity to probe participants based on their responses, which is useful when looking to identify a list of all potentially relevant outcomes that are important to different stakeholders. Additionally, a qualitative approach will help us understand why a given outcome is important to a given group of stakeholders in a more contextualized manner. This will in turn help in defining the scope of the outcomes in a manner that is most useful to various stakeholders [29].

## Study site (geographical)

The qualitative data collection will be conducted across two newborn units that will be purposively selected to provide different locations (an urban area and a rural area; this will provide perspectives from two differing populations in terms of their social economic status), disease profiles, and level of neonatal services. We will select one level 5 hospital with a high volume of neonatal care units. This hospital is located in Nairobi with approximately 66 maternity beds and a 34-bed Newborn Unit with a 10-bed Kangaroo mother care unit serving an urban population including people from informal settlements. It is a referral facility and also serves as the Teaching Hospital [30]. The other unit will be in a smaller level 4 hospital with a 20-bed newborn unit serving a more rural population and located in an area with a high prevalence of non-communicable diseases like diabetes. The diversity of these hospitals will enable us to explore a diversity of perspectives regarding outcomes across two different settings.

The study will also sample policy makers in the National Ministry of Health, which is the policy making entity for the health sector in Kenya.

## Study participants

The study participants will include newborn unit managers, frontline nursing staff and clinical teams involved in routine neonatal care service provision, parents/caregivers of babies admitted in the newborn unit during the study period, and heads of the division of neonatal and child health, division of health information systems, division of policy and research, and division of monitoring and evaluation in the Ministry of Health. Additionally, neonatal researchers and neonatologists teaching postgraduate students will also be included as they almost always constitute the panel/committee of experts that often guide policy recommendations in Kenya.

## Sample size and sampling procedures

We will use purposive sampling to access a diverse range of stakeholders, including parents, health care providers, policy makers, researchers, and professional association representatives, which will be organized as three stakeholder clusters.

1. Parents/caregivers of neonates admitted in the newborn unit,
2. Health care providers in the newborn unit (including newborn unit managers)
3. National-level policy makers (Ministry of Health heads, researchers, academics, researchers, and professional association representatives)

Table 1 summarizes the data to be collected and the anticipated sample size for each cluster. The adequacy of the sample size will be continuously evaluated during the study, and recruitment to the interviews will come to an end when the sample holds sufficient information power to develop a new knowledge base [31]. As per this model the higher the information power, the lower the sample size. Information power is a product of five factors that have an impact on sample size. These are (1) the aim of the study; (2) the specificity of the selected sample; (3) the use of established theory; (4) the quality of dialog; and (5) the analysis strategy. While the precise sample size needed for information power cannot be specified in advance of data collection and analysis, we anticipate that the proposed sample size will be sufficient based on previous qualitative research and given that the aim of this study is very specific to neonatal care within the newborn care, and participants all share experience in the day-to-day care of neonates. The quality of dialog is anticipated to be high (given previous work experience with the participants in newborn care). Feasibility is also a consideration, and we anticipate that for cluster 1, focus group discussions with 6–8 participants in each of the two hospitals (total of 12–16 participants), and for clusters 2 and 3 key informant interviews with 12 participants and 10 participants respectively as summarized in Table 1 would be feasible. This will give a total of 36–38 participants.

**Table 1:** Summary of data collection approaches and target sample size

Objective	Approach	Number to be targeted
To identify outcomes being reported in neonatal care research conducted in sub-Saharan Africa	Review of available literature for trials in neonatal clinical trials conducted in sub-Saharan countries.	N/A
To explore the important outcomes for key stakeholders in Kenya.	**Stakeholder cluster 1: parents/caregivers of children who are under neonatal care** Focus group discussions (FGDs) with parents/caregivers identified in conjunction with the nurse manager. Discussions to be conducted in English/Kiswahili depending on preference. Only those who consent to be part of the discussions	One FGD per hospital with 6 to 8 parents/caregivers (12–16 participants)
	**Stakeholder cluster 2: health care providers in the newborn unit** Key informant interview with newborn unit manager Key informant interviews with newborn unit clinicians and nurses.	*Number per hospital* 1 newborn unit manager 3 nurses per hospital (based on the three shifts per 24-h day) 1 pediatrician 1 medical officer (12 participants in total)
	**Stakeholder cluster 3: national-level policy makers** Key informant interviews (KII) with policy makers	4 policy makers at the Ministry of Health 2 neonatologists/senior pediatricians in academia 2 researchers working in neonatal research. 1 nurse from the Kenya Nursing Association 1 pediatrician from the Kenya Pediatrics Association. (10 participants)
To undertake consensus building on the important outcomes for neonatal care in Kenya.	Online/face-to-face workshop aimed at building consensus on the important outcomes identified by the different stakeholders. A presentation of the COS development process by the HIC COS and the resultant outcomes will be done during the workshop. Additionally, other outcomes identified from literature and contextual information identified through the qualitative inquiries will be presented before a facilitated consensus-building process.	20 participants in the workshop (1-day workshop) The meeting participants will be comprised of representatives from different stakeholder groups and representation from the professional associations (pediatric and nursing associations) and regulators of research in Kenya.
To assess the feasibility of collecting the proposed COS data using a routine data reporting system (Clinical Information Network).	The agreed-on outcomes will be counterchecked against the case records in two hospitals. Feasibility of collecting the outcomes on a routine electronic research database, the Clinical Information Network that collects standardized data at admission and discharge, will be explored. The congruence (or not) of the outcomes will be documented and be used to enrich the discussion and provide a snapshot of the feasibility of the health information system to collect routine data on the COS.	Two hospitals that participated in the qualitative data collection.

## Sampling procedures for the various clusters of stakeholders

### Stakeholder cluster 1: Parents/caregivers of children who are under neonatal care

We will aim to hold focus group discussions (FGDs) with 6 to 8 mothers in each of the two hospitals (total 12–16 participants). We will aim to have a mix of first-time mothers and those who have had other children. Each FGD is expected to last one and a half hours to provide ample time for all the participants to fully express their opinions about outcomes that matter to them without feeling rushed through the process.

### Stakeholder cluster 2: Health care providers in the newborn unit

Within the hospital settings, the newborn units are managed by a nursing officer but also staffed with four to 10 other nurses, at least one pediatrician, and one medical officer. We will be aiming for maximum variation in participants and as such we will purposively sample individuals for key informant interviews from each of these health care professions. Whereas it would be desirable to undertake an FGD with the nurses, it may curtail day to day running of operations in the newborn unit and as such we will also aim to undertake key informant interviews with the nurses.

The proposed hospitals are levels 4 and 5 and their staffing norms in practice include 8–12 nurses; 1–2 medical officers; 1–2 pediatricians/neonatologists; and covering the newborn unit. As such, at least 12 participants (1 newborn unit manager, 3 nurses per hospital (based on the three shifts per 24-h day), 1 pediatrician/neonatologist, and 1 medical officer) will be interviewed across the two hospitals’ newborn units. This should be feasible within the study timelines and is likely to provide data with adequate information power regarding important outcomes.

### Stakeholder cluster 3: National policy makers

For this cluster, there will be two sub-categories.

Those directly undertaking or guiding policy making at the Ministry of Health (Heads of (i) Neonatal and Child health, (ii) Health information management systems, (iii) Policy and Research, and (iv) Monitoring and evaluation) will all be invited to participate in the interviews, those who consent, will be interviewed. These are the technical leads in setting the agendas for neonatal and child health, routine data collection, research, and monitoring and evaluation.

Those who indirectly influence neonatal care policy in Kenya: researchers undertaking neonatal research, neonatologists/senior pediatricians in academia, and professional associations representatives who help guide a community of practice among their members.

The details of the sample size and data collection approaches for each stakeholder cluster are provided in Table 1.

### Participant selection and consent process

JK will engage the two newborn units through the Clinical Information Network (CIN). The purpose of the study will be explained to the hospitals’ management teams and the newborn unit managers. Thereafter an informal session will be held with all frontline workers (where possible) of the newborn units to explain the purpose of the study and engage with potential participants. Those who express an interest will be invited to take part and their informed consent sought prior to any data collection.

For the policy makers, given that they are already identified, official requests to participate in the study will be sent through the Ministry of Health processes (writing a request letter to them through the office of the Director General for Health). The officers will then be contacted for consenting and setting up of interviews.

For the researchers, those in academia, an email invite with the study information will be sent out through the Kenya Medical Research Institute and through the department of pediatrics at the universities and through the professional associations. Potential participants will be contacted via email for the consenting process and setting up of interviews at their convenience.

To ensure internal verification and validity of the study, we will audio record the interviews using an encrypted audio recording device specifically for this study. Consent to audio record will be sought before any recording is undertaken.

## Data collection methods

Data will be collected qualitatively as described below for the various stakeholder clusters.

### Stakeholder cluster 1: Parents/caregivers of children who are under neonatal care

FGDs will be undertaken to allow the collection of key information and give parents/caregivers the opportunity to raise any other issue that may be of concern to them. We will aim to have a representation of both first-time mothers and those who have had other children to improve on the variability of responses and experiences.

Compared to semi-structured interviews, FGDs enable interaction between the participants and can help to empower the participants to share insights. However, if some caregivers would like to take part but are unable or do not wish to join FGDs, we will undertake semi-structured interviews at their convenience. Semi-structured interviews have been used in other studies in similar settings [25]. A topic guide developed based on a review of the literature will be used. This guide will be piloted among parents to get their advice on the appropriateness of the questions. This will help in refining the guide prior to the data collection. It will be translated into Swahili to ensure that all parents/caregivers understand the questions being asked (see Appendix 1 (1a and 1b) for the draft questions).

Working with the newborn unit manager, we will only approach and recruit parents/caregivers of neonates who are clinically stable and close to being discharged. We will identify a venue within the hospital that is quiet and with minimal disruptions to conduct the FGDs. Two people (JK and a trained research assistant) will facilitate the discussions.

Research with parents is generally prone to power dynamics, which may inhibit the free sharing of opinions by the parents. We will minimize this by having a comprehensive introduction and consenting process where we will explain that a parent’s data will be anonymized and that the views they express will not affect the services they are receiving. Additionally, the research assistant will be someone not working in either of the hospitals to make the parents feel they can candidly share their opinions. After the first FGD, there will be a review of the findings before undertaking the second FGD to make any refinements necessary to ensure that parents feel able to share their opinions freely.

All the FGDs or interviews will be conducted with consenting participants, will be conducted in either English or Swahili, and will be in-person/face-to-face.

### Stakeholder cluster 2: Health care providers in the newborn unit and stakeholder cluster 3: national-level policy makers

For these two clusters, key informant interviews (KIIs) will be used for data collection. KIIs are more feasible than FGDs for these participants as they are logistically easier to schedule to fit with work/duty commitments. Interviews are also an appropriate method in this instance as they will allow for specific areas to be addressed, while providing flexibility to explore other areas that the interviewees may feel are important to them. This will help provide more context-specific data which will be useful in deciding whether to adopt or adapt the neonatal care COS.

The interviews will be conversational and guided by a topic guide so that all relevant points are covered (see Appendix 1 [2 and 3] for draft guide). The guide has open-ended questions and prompts to stimulate a conversational dialog and allow for unanticipated topics to be discussed. These questions will be reviewed and amended as more participants are interviewed to ensure that questions are optimized and that interviews are responsive to participants.

All the data collection tools/guides will be piloted among non-participants to ensure that the questions are fit for purpose for the various stakeholder groups. The feedback from the piloting will help further refine the tools prior to the actual field data collection. JK will employ conversational interviewing methods to undertake all the qualitative interviews to encourage the participants to provide their opinions as freely as possible. We will request permission to audio record all discussions for the purposes of transcription. JK will also maintain field notes to capture any issues that may arise during the data collection.

All the interviews will be conducted with consenting participants, will be conducted in English, and will be conducted in-person as much as possible but where need be, we will undertake them via video conferencing. The interviews will take place at a place and time that is most convenient to the participant (workplace, off-workplace, etc., as determined by them).

For each of the stakeholder clusters, towards the end of the FGD/interview, a list of outcomes from the review of literature and the HIC neonatal care set will be provided/described, and participants asked to rank them on a scale of 1 (not important) to 10 (most important).

### Consensus building workshop

After the FGDs and interviews and the ranking of the outcomes during the FGDs and interviews, a face-to-face consensus-building workshop will be held [28]. A nominal group technique (NGT)^2^ consensus meeting will be conducted with representatives from the three stakeholder clusters already involved (parents/caregivers, health care providers, and national policy makers). An NGT is preferred to allow for full contribution by all participants given the mixture of participants that will be invited for the workshop. This will enable a final consensus to be reached by various stakeholders on the core outcomes that need to be reported for neonatal care and research in Kenya. It will involve a group of 20 stakeholders (4 patient representatives, 2 Ministry of Health heads, 2 professional associations representatives, 8 health care providers, 2 newborn unit managers, and 2 researchers/academics) and will be recruited from among those who participated in the qualitative interviews where possible.

Before the start of the NGT process, presentations to the workshop participants will include an overview of the COS for use in HIC by Webbe et al. [24]; findings from the review of the literature for trials in neonatal clinical trials conducted in sub-Saharan Countries; findings of the FGD/interviews; and the ranked (by each of the three clusters) outcomes. Group discussion will then be conducted, and no outcome will be eliminated during the group discussions. Thereafter, each outcome will be rated for inclusion as a “yes” or “no”. A decision rule that has been used previously for consensus building, whereby outcomes that are rated by ≥ 70% as “yes” for inclusion will be included in the final COS and outcomes rated by < 70% will be excluded from the COS, will be used here [32]. The consensus meeting will be in person and moderated by JK and a trained research assistant.

### Feasibility of collecting the proposed COS data using a routine data reporting system

After consensus has been reached on the COS for use in neonatal care and research in Kenya, we will assess the feasibility of a routine research data reporting system in collecting the COS data.

Since this work is constrained within a PhD timeline, it may not be logistically feasible to use the routine Kenya Health Information System. We will partner with the CIN routine data collection platform that collects clinical data that span biodata, admission and discharge diagnoses, and outcomes as well as intervention-specific data that include feeding, antibiotic use, among others. We will explore the feasibility of collecting COS data routinely through a review of medical case records and structured record forms developed and refined through CIN and the Ministry of Health. CIN brings together 22 county hospitals that form the first line of referral in Kenya. It promotes the generation and use of high-quality routine information on hospital admissions to pediatric and neonatal wards as part of a learning health system [26]. CIN uses these data to promote and track adherence to guidelines and provides 3-monthly audit and feedback reports on key quality of care indicators as a means of quality improvement [27].

We will assess which of those outcomes agreed on from the consensus to be part of the COS are being documented in case records (including free text data) and if they are also being captured in the CIN database. COS captured as part of the structured record forms and in the routine CIN electronic database could form an initial set that could be integrated into the national Health Information System. Based on the findings of the analysis, each of the hospitals will be provided with tailored feedback. This can potentially improve the uptake of the COS. Tailored feedback has been shown to enhance the uptake of new guidance within LMICs [33].

## Data management

### Data storage

All interviews will be transcribed using intelligent verbatim which will enable the omittance of certain elements if they add no meaning to the script for example “ummms,” “errs,” “aahhs,” repetitions, and false starts, and transcripts will then be checked for accuracy and anonymized. Anonymization will involve the removal of all identifying information such as person and place names. JK will transcribe three interviews, to help inform the analysis process. The other audio files will be transcribed by a professional transcriber with a secure facility for the transfer of recordings and transcripts. The transcriber will sign a declaration of confidentiality.

These transcribed files will then be kept in a secure folder on a password-protected computer. All the parents/caregivers’ transcriptions will be translated into English before storage.

JK will be responsible for data collection, management, and analysis of the data. All data will be managed from an internally shared computer drive and will be stored in secure KEMRI-Welcome Trust Research Programme servers with specified collaborators provided access to the password-protected data. Data in these servers are backed up in mirror servers also within the KEMRI-Welcome Trust Research Programme. Audio files will be stored until the study is complete and all the data verified, at which point the audio files will be destroyed.

### Data analysis

This research work is based on a pragmatic paradigm whereby we seek to use the experiences of stakeholders in understanding whether a COS developed in a different setting can be adopted or needs to be adapted to the local context before it can be more useful in each setting [34]. Qualitative data will be collected to document the experiential and contextual issues relevant to informing how to adopt or adapt the COS while quantitative data will be collected to assess the capacity of the health information system to use the COS.

Qualitative data (interviews and field notes) will be typed into Microsoft Word and exported into NVIVO 11 Qualitative software (QSR International, Australia) for coding and initial analyses. Each transcript will have a unique identifier to ensure anonymity and facilitate informed analysis. We will undertake a thematic data analysis throughout the data collection period. Thematic analysis involves identifying, examining, coding, comparing, and grouping concepts to develop themes that describe the phenomenon being investigated and address the research aim [35]. JK will initially code the transcripts line-by-line and inductively code concepts relevant to the participant’s perspectives on neonatal care outcomes. This will be followed by a discussion with the collaborating team to arrive at an agreed set of themes for coding and final analysis. The write-up will be informed by qualitative research reporting guidance [36].

### Plan for communicating findings of the study

The final results will then be fed back to all study participants, managers at county and national government, and any other interested relevant party to identify possible interventions to improve use of the identified neonatal care COS.

We will further attend and make presentations in scientific conferences and public exhibitions they organized by professional associations and other entities with interests in newborn care. The international scientific community will be targeted via publications in peer-reviewed journals as well as conferences.

## Discussion

We describe a systematic approach for adopting or adapting a COS for neonatal care. We will use a mixture of methods to identify potentially relevant outcomes for consideration during the consensus meeting. We aim to include at least 20 participants in the consensus meeting including caregivers as a key stakeholder group since their perspectives might differ compared to the policy makers, researchers, hospital staff, and clinicians’ perspectives. This will ensure the validity, feasibility, and promotion of the COS use in research and routine clinical care. We do recognize that it may be difficult to all the relevant stakeholders, but for transparency, we have selected participants for the consensus meeting with a clear rationale for each.

One of the limitations for this work is that we will not include how to measure the outcomes agreed on during the consensus meeting. Future work will likely focus on how to measure the outcomes and assess their usage by using a before and after study design once the COS has been introduced in the system.

This COS will help outcome data comparison and will enable adequate and efficient comparison of treatment strategies using research data and also using routine data. In addition, this work will describe a methodology which entities can use to adopt or adapt COS developed in a different setting. This will contribute to high-quality evidence in neonatal care and also reduce research wastage.

## Status

As of the submission time of this protocol, outcome identification from the rapid review of literature has been completed, qualitative data collection and analysis is ongoing, and participants for the consensus meeting have been identified. The target is to finalize qualitative data collection by 30 June 2023 and undertake the consensus meeting on 21 July 2023.

### Supplementary Information

**Additional file 1. Interview guides.**

## Data Availability

All study data will be anonymized and kept confidential and for the parents/caregiver’s consent forms translation to Swahili will be undertaken.

## Appendix 1: Interview guides

### Interview guides

#### 1a. Parent/caregiver interview guide (English version)

##### Engagement questions

When did your baby get admitted to the unit?

What has happened since the child got admitted? (Probe for parent and child)

##### Explorative questions

How do you judge how well your baby is doing?

How do you think the doctors and nurses judge how well your baby is doing?

What makes you concerned about your baby’s health? (Probe for things they’ve been worried about)

What are your expectations/concerns about your baby when you go back home and as they grow?

Provide definition of an outcome using an example in plain language (guided by COMET initiative plain language summary).

What would you say are the most important outcomes for your baby to consider effective care/treatment?

Describe the outcomes from the HIC neonatal care set and ask them to rank the outcomes in order of their importance to them. (1 [least important] to 10 [most important])

##### Exit question

Is there anything else you would like to say in relation to outcomes of your baby?

#### 1b. Parent/caregiver interview guide (Swahili version)

Maswali ya ufunguzi

Je, mtoto wako alilazwa lini kwenye hospitali?

Nini kimemfanyika tangu mtoto alazwe? (Peleleza kwa yalitokea kwa mzazi na pia kwa mtoto)

Maswali ya uchunguzi

Je, unaamuaje ama unajuaje kama mtoto anapata nafuu?

Unafikiri madaktari na wauguzi wanajuaje kama mtoto wako anapata nafuu?

Ni nini kinachokufanya uwe na wasiwasi kuhusu afya ya mtoto wako? (Peleleza mambo ambayo wamekuwa na wasiwasi nayo)

Una matarajio yapi au wasiwasi upi kuhusu mtoto wako unaporudi nyumbani na anapo endelea kukua?

Toa ufafanuzi wa maana ya matokeo kwa kutumia mfano katika lugha ya kawaida (inayoongozwa na muhtasari wa lugha ya kawaida ya COMET).

Je, unaweza kusema ni matokeo yepi muhimu zaidi kwa mtoto wako ili eweze kusema umepata huduma bora katika hii hospitali?

Eleza matokeo kutoka kwa seti ya matokeo kwenye tafiti za utunzaji wa watoto wachanga katika nchi zenye mapato ya juu na uwaombe kuorodhesha matokeo hayo kulingana na umuhimu kwao. (1 [umuhimu wa chini] hadi 10 [muhimu zaidi])

Swali la kumalizia

Je, jambo jingine lolote ungependa kusema kuhusiana na matokeo ya mtoto wako?

#### 2. Key informant interview guide for health care providers in the hospitals

Tell me about day-to-day activities in the neonatal unit.

For how long have you been working in the neonatal unit?

When a newborn is admitted here what happens? (probe for the admission process including communication to the caregiver/parent

Can you describe the work environment? (probe for availability supplies and commodities; data capture tools etc.?

Provide definition of an outcome using an example in plain language (guided by COMET initiative plain language summary).

What would you say are the most important outcomes for a neonate admitted in the newborn unit? (Probe why they think each is important)

Do you collect the data on the outcomes mentioned above routinely? (probe for measurement of the outcomes)

Describe outcomes from the HIC neonatal COS and ask them to rate or rank them in order of importance (1 [least important] to 10 [most important])

##### Provide brief description of a COS

What are your perceptions about having a COS for use in your area of work (Probe for perceptions on research and on routine work)

Exit Question

Is there anything else you would like to say in relation to outcomes in neonatal care?

#### 3. Key informant interview guides for national policy makers

Tell me about your day-to-day activities (probe for activities linked to neonatal health)

What are the most important outcomes that have been prioritized neonatal health? (probe for the process of generation of the outcomes and their implementation, measurement and evaluation)

How often is the data from the routine system and or research inform development of policies (probe for the process of data use; how it has worked; what can be changed)

Describe outcomes from the HIC neonatal COS and ask them to rank them in order of importance ((1 [least important] to 10 [most important])

##### Provide brief description of a COS

A COS is an agreed minimum set of outcomes to be measured and reported in all trials in a specific area like newborns admitted to the hospital.

What are your reactions and thoughts about establishing a COS for neonatal care?

What are the potential benefits/ (or drawbacks)?

Can you suggest key criteria an outcome must fulfill to be included as a “core outcome” — why? (Probe for feasibility of its measurement, clinically relevant, scope)

What do you think needs to be considered in implementing the COS?

What impacts (e.g., research, practice, policy, patient outcomes) do you think the implementation of a COS will have?

What would it take to include an outcome in the routine data collection systems (probe for frequency of review of data collection indicators, process of change, cost implication)?

Exit question

Is there anything else you would like to say in relation to outcomes in neonatal care?

## Footnotes

1 A sequential process through which the opinions of participants, for example on outcomes identified from a systematic review of the literature, are sought anonymously. Participants do not interact directly; rather, after the completion of each round of questionnaires, the collated group responses are fed back to participants before the next round. This reduces the risk of domination of the process by an individual(s).

2 Nominal group technique is a structured method for group brainstorming that encourages contributions from everyone and facilitates quick agreement on the relative importance of issues. Team members begin by writing down their ideas, then selecting which idea they feel is best. Once team members are ready, everyone presents their favorite idea, and the suggestions are then discussed and prioritized by the entire group using a point system.
